# Quercetin-treated rat sperm enables refrigerated transport with motility and fertility for five days

**DOI:** 10.1038/s41598-021-02166-6

**Published:** 2021-11-22

**Authors:** Katsuma Yamaga, Satohiro Nakao, Nobuyuki Mikoda, Hidetaka Yoshimoto, Ena Nakatsukasa, Naomi Nakagata, Toru Takeo

**Affiliations:** 1 Division of Reproductive Engineering, Center for Animal Resources and Development (CARD), Institute of Resource Development and Analysis, Kumamoto University, 2-2-1 Honjo, Chuo-ku, Kumamoto, 860-0811 Japan; 2 Division of Reproductive Biotechnology and Innovation, Center for Animal Resources and Development (CARD), Institute of Resource Development and Analysis, Kumamoto University, 2-2-1 Honjo, Chuo-ku, Kumamoto, 860-0811 Japan; 3 Department of Animal Model Development, Brain Research Institute, Niigata University, 1-757 Asahimachidori, Chuo-ku, Niigata, 951-8585 Japan

**Keywords:** Biological techniques, Developmental biology, Zoology

## Abstract

Shipment of laboratory rats between animal facilities is frequently performed using special containers. However, the shipment of live animals is associated with potential risks of infectious diseases, escape and death during shipment and animal welfare issues. The transport of cold-stored sperm avoids such risks; however, there have been no reports on cold storage of rat sperm. We previously reported that dimethyl sulfoxide (DMSO) and quercetin maintained the motility and fertilising abilities of cold-stored mouse sperm stored for 10 days. The present study investigated the efficacy of DMSO and quercetin in the cold storage of rat sperm. Quercetin maintained motility and fertility of cold-stored rat sperm stored for 5 days. After in vitro fertilisation using cold-stored sperm, pronuclear and two-cell embryos developed normally to pups following embryo transfer. Therefore, we demonstrated that live pups could be obtained from sperm transported using the cold-storage system. We conclude that cold storage of rat sperm may provide an efficient system for transporting rat resources as an alternative to shipping live animals.

## Introduction

Genetically engineered rats are frequently transported between animal facilities in special containers. However, the shipment of live animals is associated with potential risks, such as spreading infectious diseases, escape and death of animals and animal welfare issues. On the other hand, the transport of cryopreserved sperm and embryos could be a useful means to avoid such risks. The cryopreservation of sperm and embryos requires ultra-low temperatures using special containers (dry shippers) and proficient skills to handle the cryopreserved samples^1,2^. Furthermore, the use of freeze-dried sperm in the production of pups via intracytoplasmic sperm injection is associated with technical barriers^3^. Simple and user-friendly techniques are required to transport genetically engineered rats to overcome these issues.

The transport of cold-stored sperm is potentially useful for the shipment of mutant rats. However, the motility of cold-stored rat sperm rapidly decreases and there may be damage to the acrosomal membrane and DNA in Tyrode lactate-HEPES, Ham’s F10 medium plus raffinose or trehalose or fructose, tris (hydroxymethyl) aminomethane (Tris)–citrate, Krebs–Ringer bicarbonate, lactose-egg yolk and phosphate-buffered saline at 4 °C within 24 h^4–6^. We previously developed a cold-storage system for mouse sperm^7^. Mouse sperm stored in cold-storage medium of Lifor supplemented with dimethyl sulfoxide (DMSO) and quercetin at 4 °C maintained motility and fertility for 10 days^8^. The protective effects of DMSO and quercetin may be applicable to rat sperm; however, there have been no previous reports about the cold-storage of rat sperm or in vitro fertilisation (IVF) using cold-stored rat sperm.

The present study examined the efficacy of cold-storage systems for rat sperm. We first examined the effects of quercetin and DMSO on the motility and fertilisation ability of cold-stored sperm. We then examined the fertilisation capacity of sperm stored in cold-storage medium containing DMSO and quercetin for different storage periods. Next, we examined the developmental ability of embryos obtained by IVF using sperm cold-stored with DMSO and quercetin. Finally, we studied the effects of transportation on cold-stored cauda epididymides as well as the fertilisation and developmental abilities of cold-transported rat sperm.

## Results

### DMSO and quercetin improved the motility of cold-stored rat sperm

**Figure 1:**
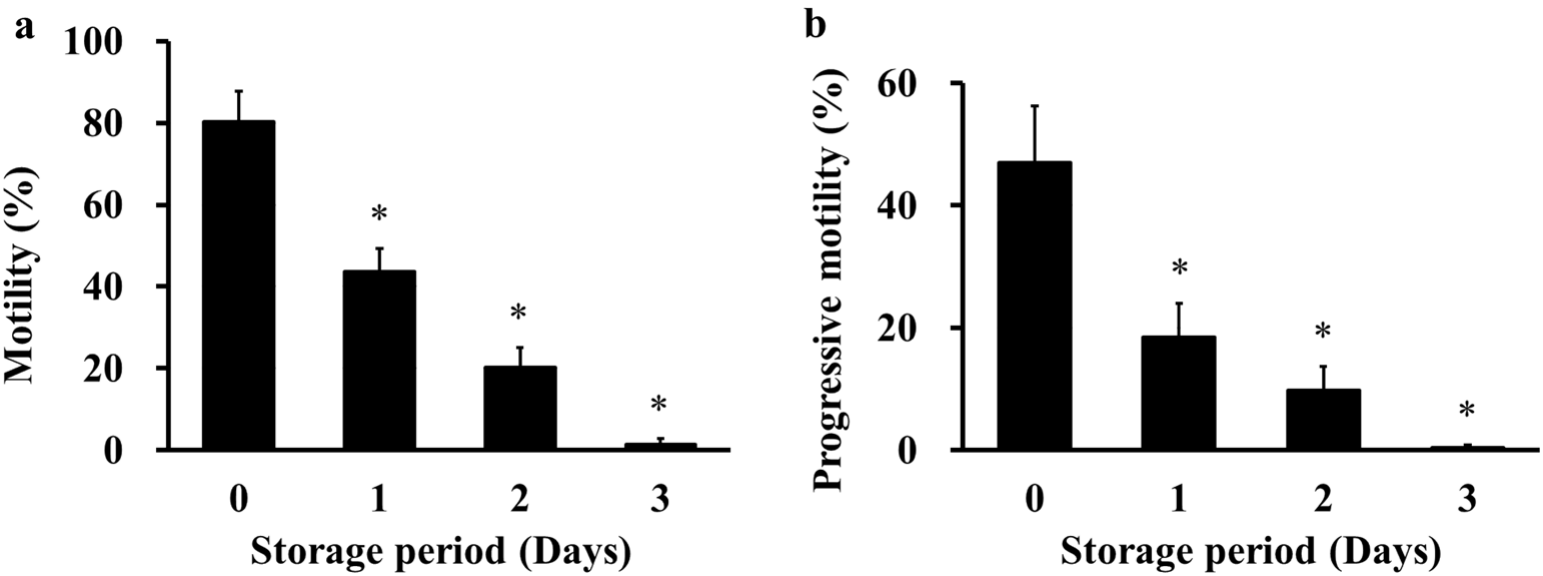
Effect of storage period on motility parameters of cold-stored sperm. Cauda epididymides were cold stored in Lifor at 4 °C for 0–3 days. After cold storage, sperm were cultured in mHTF for 2 h and motility was analysed using an IVOS sperm analyser. Motility was measured as the percentage of sperm moving at a speed of ≥ 5 µm/s (**a**). Progressive motility was measured as the percentage of sperm moving at ≥ 50 µm/s with a progressiveness of > 50% (**b**). Results are expressed as mean ± SD (n = 3–4 male rats/group, 14 male rats were used). *P < 0.05 compared with day 0.

**Figure 2:**
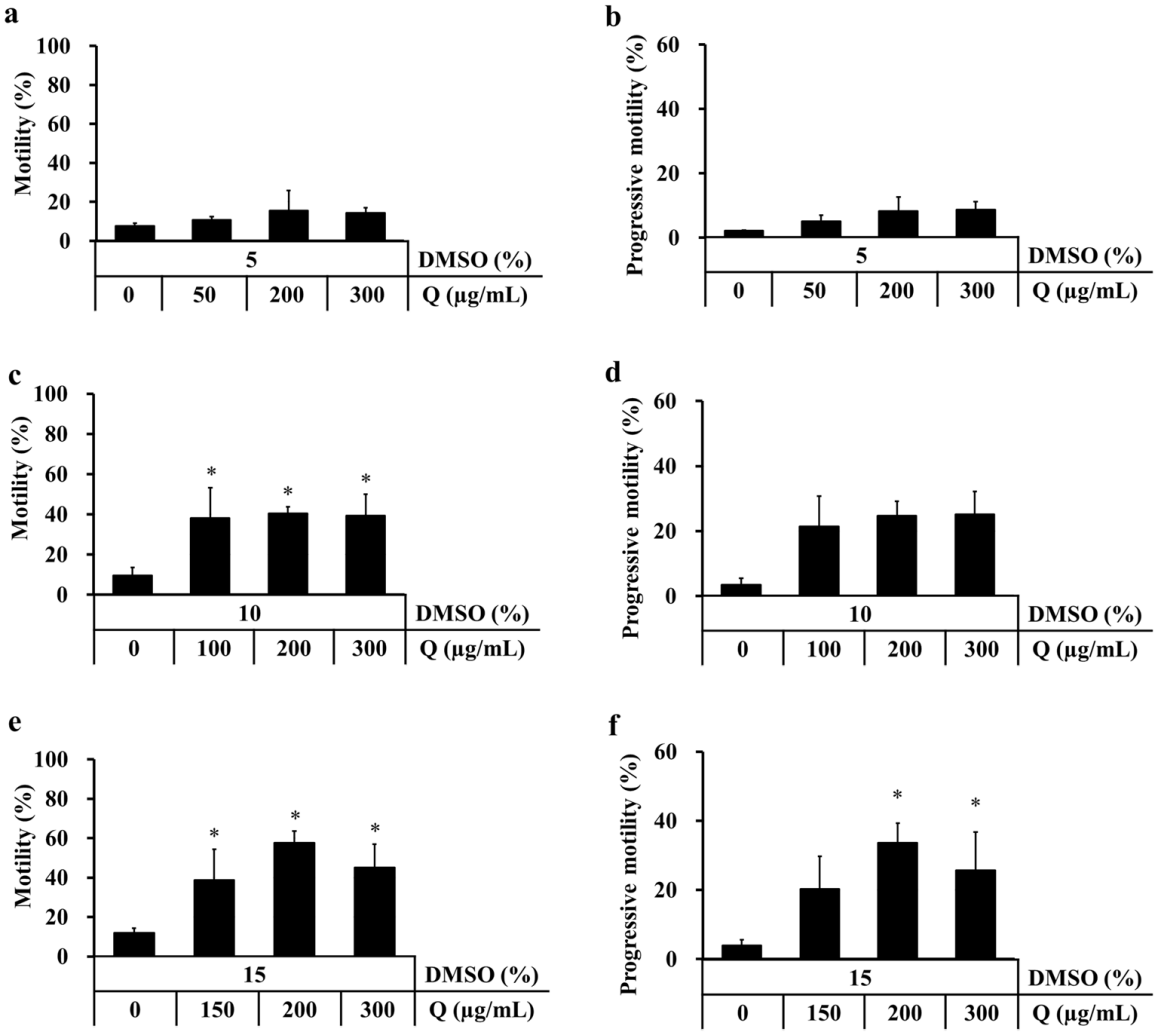
Effect of different concentrations of DMSO and quercetin (Q) on motility of cold-stored sperm. Cauda epididymides were cold-stored in Lifor with various concentrations of DMSO (5%, 10% and 15%) and quercetin (0, 50, 100, 150, 200 and 300 µg/mL) at 4 °C for 3 days. After cold-storage, sperm were cultured in mHTF for 2 h and motility was analysed using an IVOS sperm analyser. Motility was measured as the percentage of sperm moving at a speed of ≥ 5 µm/s (**a**,**c**,**e**). Progressive motility was measured as the percentage of sperm moving at ≥ 50 µm/s and progressiveness of > 50% (**b**,**d**,**f**). Results are expressed as mean ± SD (n = 3–5 male rats/group, 45 male rats were used).*P < 0.05 compared with 0 µg/mL quercetin in each experiment.

Sperm motility was analysed in sperm cold-stored for 3 days in a cold-storage solution containing various concentrations of DMSO and quercetin to determine their optimal concentrations. We first confirmed how long cold-stored rat sperm could be stored in the cold-storage medium of Lifor and maintain motility. The motility and progressive motility of cold-stored rat sperm stored in Lifor decreased after 1 day (Fig. 1a,b). However, the motility of the cold-stored sperm did not change when stored in Lifor containing 50–300 µg/mL quercetin in 5% DMSO (Fig. 2a,b). Concentrations of 100–300 µg/mL quercetin in 10% DMSO increased the percentage motility (Fig. 2c). The progressive motility of the cold-stored sperm did not change with the addition of 100–300 µg/mL quercetin in 10% DMSO (Fig. 2d). Sperm stored in Lifor containing 150–300 µg/mL quercetin in 15% DMSO showed increased motility (Fig. 2e). Furthermore, the progressive motility increased with 200 and 300 µg/mL quercetin in 15% DMSO (Fig. 2f). The highest rate of motility was observed with Lifor containing 200 µg/mL quercetin in 15% DMSO.

### Cold-storage of rat sperm in 15% DMSO and 200 µg/mL quercetin improved the fertilisation rate

**Figure 3:**
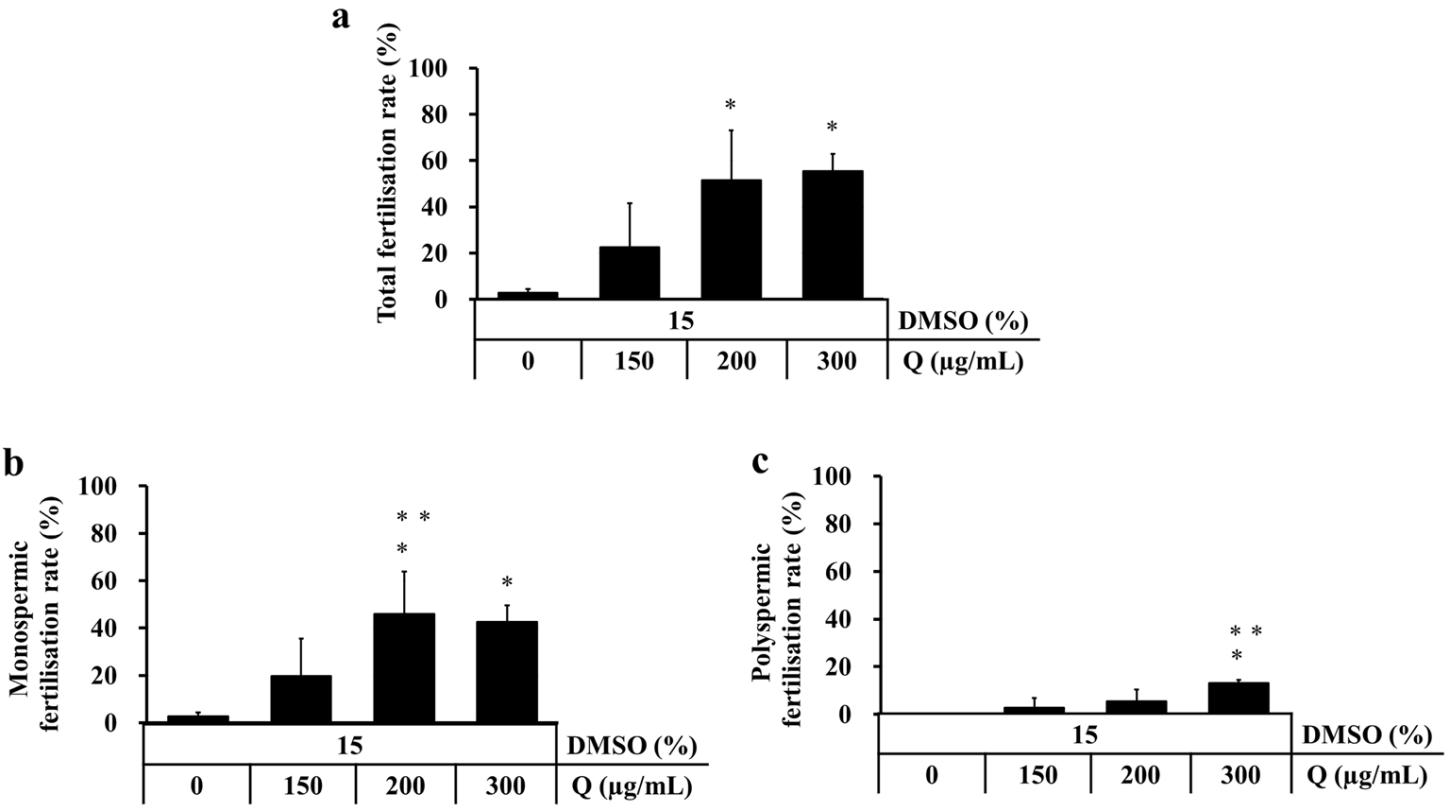
Effect of different concentrations of DMSO and quercetin (Q) on fertility of cold-stored sperm. Cauda epididymides were cold-stored in Lifor with 15% DMSO and various concentrations of quercetin (0, 150, 200 and 300 µg/mL) at 4 °C for 3 days. After cold-storage, sperm were cultured in mHTF and cumulus-oocytes complexes collected from a female rat were introduced into the sperm suspension. The total fertilisation rate was calculated as the number of fertilised oocytes divided by the total number of oocytes and multiplied by 100 (**a**). The monospermic fertilisation rate was calculated as the number of monospermic oocytes (two pronuclei and sperm tail or sperm tail in the cytoplasm) divided by the total number of oocytes and multiplied by 100 (**b**). The polyspermic fertilisation rate was calculated as the number of polyspermic oocytes (more than three pronuclei or two sperm tails in the cytoplasm) divided by the total number of oocytes and multiplied by 100 (**c**). Results are expressed as mean ± SD (n = 3–7 male rats/group, 20 female rats and 20 male rats were used). *P < 0.05 compared with 0 µg/mL quercetin. **P < 0.05 compared with 150 µg/mL quercetin.

IVF was performed using sperm cold-stored for 3 days with Lifor containing 15% DMSO and different concentrations of quercetin to examine the effect of DMSO and quercetin on the fertilising ability of cold-stored sperm. Sperm stored with Lifor containing 200 and 300 µg/mL quercetin showed higher rates of total and monospermic fertilisation than sperm stored with Lifor containing DMSO only or 150 µg/mL quercetin (Fig. 3a,b). Rate of polyspermic fertilisation was increased at 300 µg/mL quercetin in 15% DMSO (Fig. 3c). Therefore, 200 µg/mL quercetin in 15% DMSO was used for the following experiments based on the results of motility and fertility analysis.

### Cold-stored sperm maintained fertilising ability for up to 5 days

**Figure 4:**
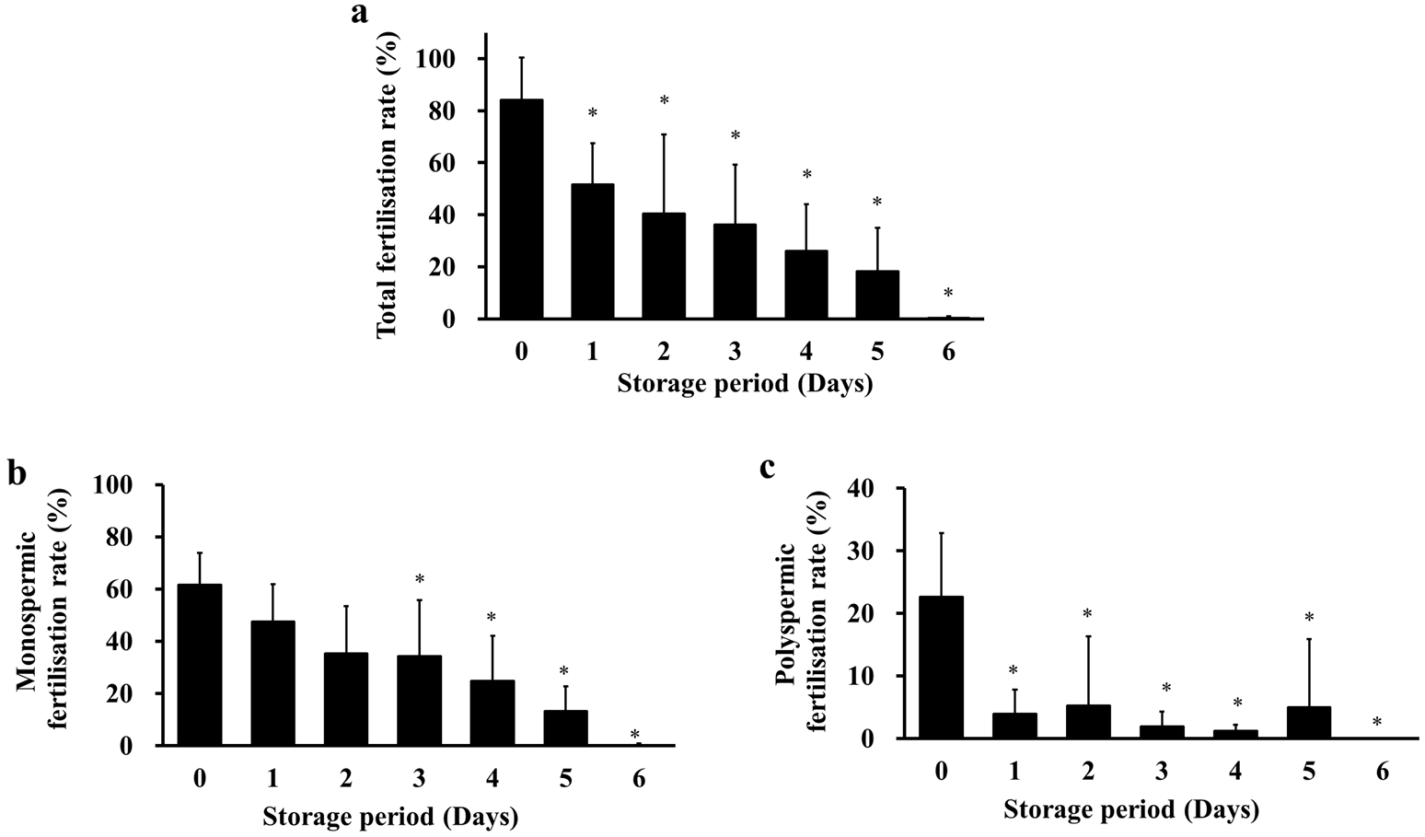
Effect of storage period on the fertilisation ability of cold-stored sperm. Cauda epididymides were cold-stored in Lifor containing 15% DMSO and 200 µg/mL quercetin at 4 °C for 0–6 days. After cold-storage, sperm were cultured in mHTF and cumulus-oocytes complexes collected from a female rat were introduced into the sperm suspension. The total fertilisation rate was calculated as the number of fertilised oocytes divided by the total number of oocytes and multiplied by 100 (**a**). The monospermic fertilisation rate was calculated as the number of monospermic oocytes (two pronuclei and a sperm tail or a sperm tail in the cytoplasm) divided by the total number of oocytes and multiplied by 100 (**b**). The polyspermic fertilisation rate was calculated as the number of polyspermic oocytes (more than three pronuclei and more than two sperm tails or more than two sperm tails in the cytoplasm) divided by the total number of oocytes and multiplied by 100 (**c**). Results are expressed as mean ± SD (n = 4–8 male rats/group, 46 female rats and 46 male rats were used). *P < 0.05 compared with day 0.

IVF was performed using sperm cold-stored for 0–6 days to examine the effect of storage period on fertilisation rate. Fertilisation rates gradually decreased after 1 day, but fertilised oocytes were obtained from cold-stored sperm for up to 5 days (Fig. 4a). The monospermic fertilisation rate of fresh and cold-stored sperm was stable for up to 2 days and decreased after 3 days (Fig. 4b). Rates of polyspermic fertilisation of cold-stored sperm were lower than that of fresh sperm. (Fig. 4c).

**Figure 5:**
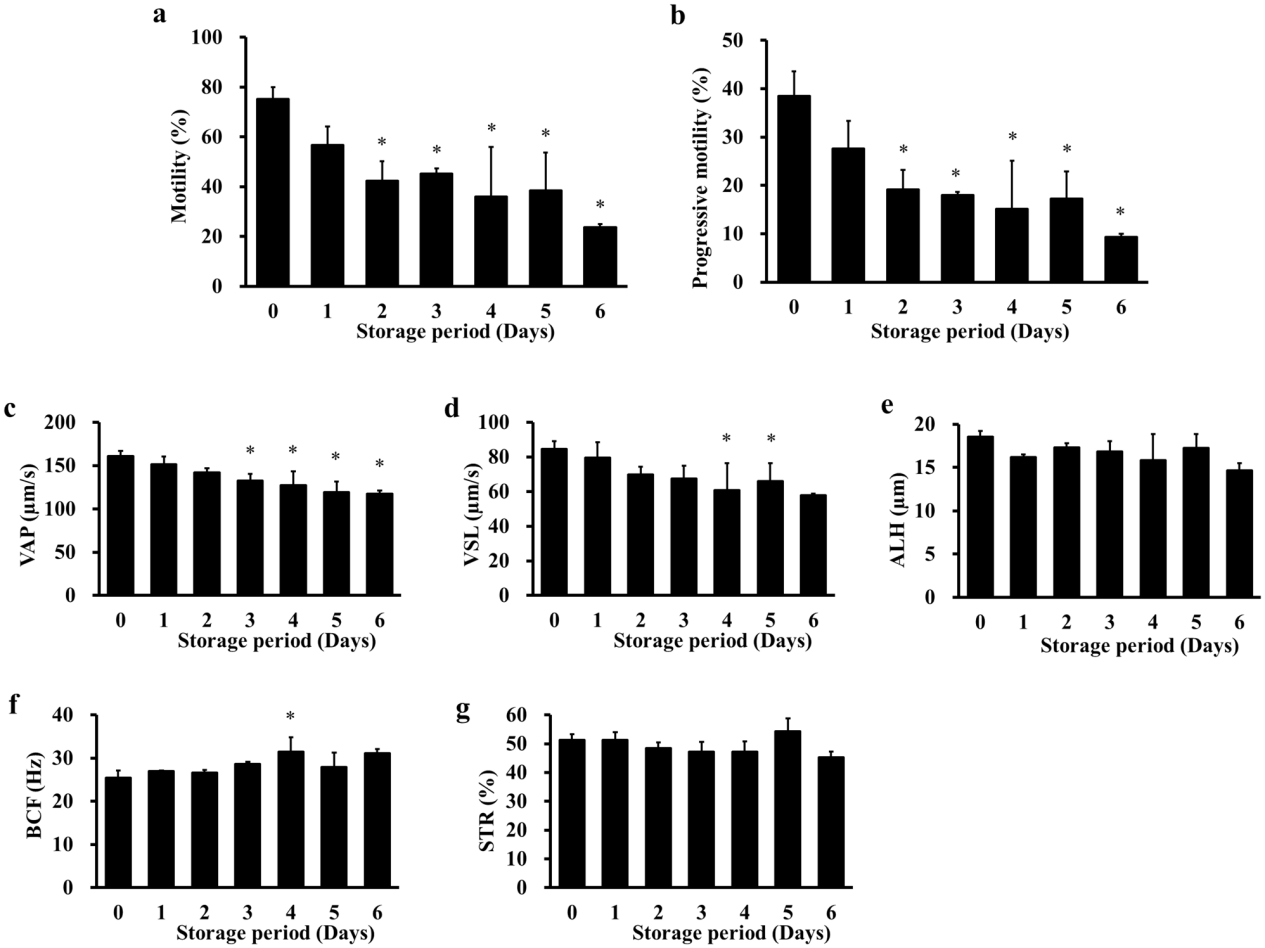
Effect of storage period on the motility of cold-stored sperm. Cauda epididymides were cold-stored in Lifor containing DMSO (15%) and quercetin (200 µg/mL) at 4 °C for 0–6 days. After cold-storage, sperm were cultured in mHTF for 6 h and the motility was analysed using an IVOS sperm analyser. The following motility parameters were measured: motility (**a**) and progressive motility (**b**), VAP (**c**), VSL (**d**), ALH (**e**), BCF (**f**) and STR (**g**). Results are expressed as mean ± SD (n = 3–5 male rats/group, 26 male rats were used). *P < 0.05 compared with day 0.

Motility and progressive motility decreased after 2 days (Fig. 5a,b). Furthermore, the average path velocity (VAP, which shows the average velocity of motile sperm) decreased after 3 days (Fig. 5c). VSL decreased for 4–5 days (Fig. 5d). BCF increased for 4 days (Fig. 5f). ALH and STR were not changed (Fig. 5e,g).

### Embryos derived from cold-stored rat sperm developed into live pups

Embryo transfer was performed to evaluate the developmental ability of embryos produced by IVF using fresh and cold-stored sperm (after a cold-storage period of 3 days). Embryos derived from cold-stored sperm developed normally into live pups (Table 1).

**Table 1:** Birth rate of fresh and cold-stored rat sperm.

Storage period (day)	No. of recipients	No. of transferred fertilised oocytes	No. of pups (%)
0	3	60	20 (33.3 ± 8.5)
3	3	52	17 (32.8 ± 6.1)

In the IVF, 6 female and 6 male rats were used.

Ten vasectomised male rats and 6 pseudo-pregnant rats were used for embryo transfer.

### Embryos derived from cold-transported sperm developed into live pups

**Figure 6:**
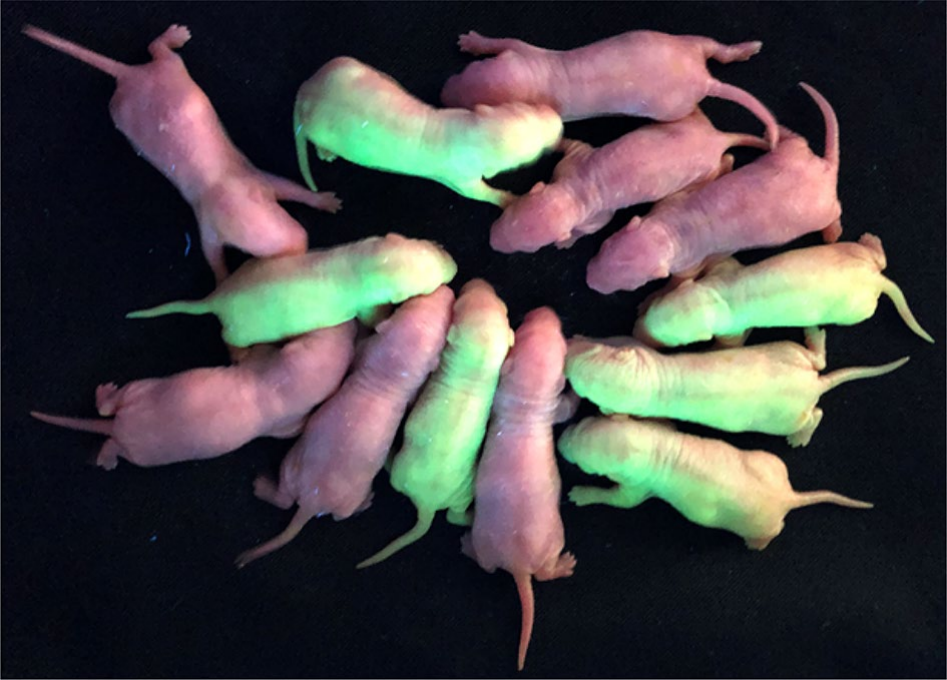
Live pups derived from in vitro fertilisation using cold-transported rat sperm. Fertilised oocytes were transferred into the oviducts of pseudo-pregnant female rats. The embryos developed normally into live pups 22–23 days after embryo transfer. Wild type and GFP-positive pups are shown.

IVF and embryo transfer using cold-transported EGFP-labelled rat sperm were performed to confirm whether sperm retained normal fertilisation and developmental abilities after cold-storage transport from Niigata University to Kumamoto University. Fertilised oocytes were obtained by IVF and the embryos developed normally into live pups (Table 2, Fig. 6).

**Table 2:** Fertilisation rate and birth rate of cold-transported rat sperm.

Experiment no	No. of inseminated oocytes	No. of fertilised oocytes (%)	No. of recipients	No. of transferred fertilised oocytes	No. of pups (%)	No. of GFP positive pups (%)
1	112	47 (41.2 ± 21.9)	1	24	17 (70.8)	6 (35.3)
2	160	73 (44.8 ± 25.3)	1	20	13 (65.0)	6 (46.2)
Total	272	120 (44.1 ± 23.7)	2	44	30 (68.1)	12 (40.0)

In the IVF, 6 female and 2 male rats were used.

Ten vasectomised male rats and 2 pseudo-pregnant rats were used for embryo transfer.

## Discussion

The addition of DMSO and quercetin to cold-storage medium prolonged the motility and fertility of cold-stored rat sperm. Cold-stored rat sperm maintained its fertilising ability for 5 days in vitro. Embryos produced by IVF using the cold-stored rat sperm developed normally to pups following embryo transfer. Furthermore, cold-transported sperm maintained fertility and developmental abilities. This cold-storage technique can be used for the simple and efficient transport of sperm from genetically modified rats.

The present study demonstrated the successful transfer of sperm to a different animal facility and the production of live pups via IVF and embryo transfer from cold-transported rat sperm stored for 64 h. The storage period of cold-stored rat sperm was limited to 5 days, which would be applicable to the domestic or international shipment of genetically modified rats. We recommend shipment on a Monday, with IVF and embryo transfer complete by Friday.

The composition of the storage medium is critical to the yield of embryos after cold storage of rat sperm. Cold-storage medium containing 10% DMSO and 200 µg/mL quercetin maintained the fertility of rat sperm for 5 days (Fig. 3). We previously reported that cold-storage of mouse sperm in medium containing 10% DMSO and 100 µg/mL quercetin maintained fertility for 10 days^8^. The protective effects of DMSO and quercetin were previously shown to include alleviation of oxidative stress in rooster and rabbit sperm stored at 4 °C and 5 °C^9,10^. In mouse sperm, quercetin was localised in the mitochondria and maintained mitochondrial activity during cold preservation^8^. We assumed that the protective effects of quercetin would be similar in cold-stored rat sperm by reducing oxidative stress and maintaining mitochondrial activity.

DMSO showed a protective effect in cold-stored mouse sperm, whereas it showed no effect in cold-stored rat sperm. DMSO is a polar non-protic solvent that can react with and remove hydroxyl radicals^11^. Excessive production of hydroxyl radicals (a reactive oxygen species) can damage cell membranes and mitochondria, leading to decreased sperm motility^12^. However, adverse effects of DMSO were reported on the motility and integrity of the membranes and acrosome of rat sperm at room temperature^13^. Therefore, further studies are required to identify the optimal solvents as alternatives to DMSO to improve the quality of cold-stored rat sperm.

The total fertilisation rates of rat sperm cold-stored for 1–5 days were lower than that of fresh sperm (84.2%). On the other hand, the rates of polyspermic fertilisation of cold-stored sperm were lower (1.2–5.2%) than those of fresh sperm (22.7%). In a previous study, polyspermic fertilisation was observed in CD (29%) and Long-Evans (20%)^14^ rats. Polyspermy was common in rat IVF using fresh sperm; however, the use of cold-stored rat sperm reduced the rate of polyspermic fertilisation. We assumed the difference in motile activity between fresh and cold-stored rat sperm affected the rate of polyspermic fertilisation. Polyspermy is inhibited by the hardening of the zona pellucida with the surface reaction of the cell membrane of the ooplasm after membrane fusion with sperm and oocyte^15^. Exocytosis of cortical granules and modification of the zona pellucida occur within a few minutes after fertilisation^16^. After exocytosis of the cortical granule, ovastacin and spark inhibited sperm binding to zona pellucida within a few minutes to 30 min^17–22^. We observed many sperm in the perivitelline space during IVF using fresh sperm, whereas a few sperm existed in the perivitelline space during IVF using cold-stored sperm. Reduced motility and progressive motility of cold-stored sperm may delay the penetration of second or later sperm through the zona pellucida, avoiding polyspermic fertilisation.

Delayed penetration of cumulus cells also reduces polyspermic fertilisation in cold-stored rat sperm. Cumulus cells were dispersed by fresh sperm at 6 h after insemination, whereas cumulus cells remained with cold-stored sperm. Thus, cold-stored sperm may take longer than fresh sperm to penetrate the layer of cumulus cells. The delayed penetration of cold-stored sperm could be attributed to reduced motility or delayed or dysfunction of the acrosome reaction. Acrosin is an enzyme released from the acrosome during the acrosome reaction^23,24^. Sperm from acrosine-deficient rats passed through the zona pellucida to be fertilised, but had difficulty penetrating the cumulus cells^25–27^. Cold-induced acrosine dysfunction may be related to the delayed penetration of cumulus cells in cold-stored sperm. In addition, the delayed penetration of sperm allows sufficient time to react to the modifications of the zona pellucida and positively reduce polyspermic fertilisation in cold-stored sperm. Further studies are required to elucidate the mechanisms involved.

Cold-transport technology provides a practical and efficient method to transport genetically modified rats. In mice, domestic and international transportation have been successfully carried out using the cold-storage technique. Cold transport of rat sperm would be advantageous because rats are difficult to transport as they are more sensitive to environmental changes during transportation and larger than mice^28,29^. Cheaper shipment costs of cold-stored rat sperm would be a beneficial alternative to the shipment of live animals. Some limitations of cold-storage technique about storage condition at refrigerated temperatures and period for 5 days have remained.

Genome editing technology enhances the value of laboratory rats as important models of human diseases and drug development in physiology and toxicology^30^. As a consequence the production and transport of genetically modified rats will increase over time. We believe the improving the transport system of rat resources using the technology will accelerate multi-institutional collaboration, improve reproducibility of animal science and promote further innovations in biomedical science.

## Methods

### Animals

Jcl:SD rats were purchased from CLEA Japan Inc. (Tokyo, Japan) to use as sperm and oocyte donors. Sperm were obtained from male rats (11–15 weeks old) and oocytes were obtained from immature female rats (5–6 weeks old). Crlj:CD(SD) male rats (> 13 weeks old) were purchased from Charles River Japan (Kanagawa, Japan) to use as vasectomised male rats for embryo transfer. Crl:CD(SD) female rats (10–12 weeks old) were purchased from Charles River Japan to use as recipients for embryo transfer. Male transgenic rats (SD-Tg(CAG-EGFP)4Osb) and female rats (Slc:SD) purchased from Slc Japan (Shizuoka, Japan) were used for cold transport. The breeding environments were light (07:00–19:00 h) and dark (19:00–07:00 h), room temperature was 22 °C ± 2 °C and with free access to food and water. Male rats were kept in two animals per cage, and female rats were kept in three animals per cage. The Animal Care and Use Committee of Kumamoto University approved the protocols for animal experiments (ID: A2021-025), and all methods were performed in accordance with ARRIVE guidelines and relevant and regulations.

### Media and reagents

Lifor was used as cold-storage medium for cauda epididymides (Lifor, Detraxi Inc., boca Raton, Florida, USA). DMSO (FUJIFILM Wako Pure Chemicals Co., Osaka, Japan) was dissolved in Lifor to final concentrations of 5–20% (v/v). Quercetin (FUJIFILM Wako Pure Chemicals Co., Osaka, Japan) was dissolved in DMSO to final concentrations of 50–300 µg/mL. Modified human tubal fluid (mHTF) was used as sperm collection and IVF medium^31,32^. All media were covered with paraffin oil (Nacalai Tesque Inc., Kyoto, Japan) and aerated with 5% CO_2_ at 37 °C prior to use. Three types of mixed aesthetic agents were prepared by mixing midazolam (2 mg/kg), medetomidine (0.375 mg/kg) and butorphanol (2.5 mg/kg).

### Cold storage of cauda epididymides

Cauda epididymides were removed from mature male rats euthanised by cervical dislocation and cauda epididymides were transferred to 1.5-mL plastic tubes containing 1 mL of cold-storage solution (Lifor + DMSO + quercetin). The tubes were placed in a paper box with a digital thermometer and date logger (Thermochron iButton; Maxim Integrated Products, Inc.). The box was placed in a vacuum bottle (JMK-500, Thermos Co., USA) with two cold packs (60 mm × 180 mm) precooled in a refrigerator at 4 °C. The bottle was placed in a Styrofoam box (205 × 125 × 130 mm^3^) with four cold packs (140 mm × 250 mm) precooled in a refrigerator at 4 °C. The packed Styrofoam box stored in a refrigerator at 4 °C for 0–6 days. The temperature was measured with a thermometer and was found to be 2–5 °C.

### Preparation of sperm

After the cold storage, the cauda epididymides were removed from cold storage solution and the excess solution was gently wiped off with filter paper. Cauda epididymides were washed in saline and transferred to paraffin oil of sperm collection dish. In the paraffin oil, cauda epididymides were made a short incision using microdissecting scissors and sperm were transferred into a 400-µL drop of mHTF using glass rod (15 mm). The sperm suspension was incubated in an incubator at 37 °C with 5% CO_2_. Sperm concentration was calculated using a hemocytometer (Erma, Tokyo, Japan) and sperm suspensions were added to a drop of 200 μL IVF medium covered with paraffin oil (the final concentration was 500–1500 sperm/µL). Sperm were incubated at 37 °C and 5% CO_2_ for 2 h and used for IVF and assessment of sperm motility.

### In vitro fertilisation

Immature female rats were injected with 30 IU equine chorionic gonadotropin (PMSG, ASKA Animal Health Co. Ltd, Japan). At 50–55 h after PMSG injection, the rats were injected with 30 IU human chorionic gonadotropin (hCG, ASKA Animal Health Co. Ltd, Japan). At 16–18 h post-administration of hCG, immature females were euthanised by cervical dislocation and their oviducts were collected. The oviducts were transferred in paraffin oil of the IVF dish containing a sperm suspension. Cumulus oocyte complexes were collected from the ampulla of the oviduct into the IVF drop. The oocytes and sperm were incubated at 37 °C with 5% CO_2_ for 6–7 h. The oocytes were then washed three times with 80 µL of mHTF drops covered with paraffin oil and cultured at 37 °C with 5% CO_2_. The oocytes were observed under an inverted microscope 23–24 h after insemination and the fertilisation rate was calculated as the number of fertilised oocytes (monospermic oocytes that showed two pronuclei and a sperm tail or a sperm tail in the cytoplasm and polyspermic oocytes with more than three pronuclei and more than two sperm tails or more than two sperm tails in the cytoplasm were determined to be fertilised oocytes) divided by the total number of oocytes (unfertilised oocyte and fertilised oocyte) and multiplied by 100.

### Assessment of sperm motility

The motility of fresh and cold-stored sperm was evaluated using a computer-assisted sperm analyser (IVOS Sperm Analyzer, Hamilton-Thorne Research Co. Ltd., USA). Fresh sperm were diluted to a final concentration of 500 sperm/µL and cultured in 200 µL of mHTF drops covered with paraffin oil for 2 h at 37 °C and 5% CO_2_. Next, 10 µL of the sperm suspension was collected and added to the measurement chamber to measure motility and other parameters. Sperm motility was calculated as the ratio of sperm that moved at a speed of ≥ 5 µm/s. Progressive motility was calculated as the percentage of total sperm that progressed ≥ 50 μm/s and had a progressiveness ratio of ≥ 50%. VAP was calculated as the average velocity of the motile sperm. Progressive velocity (VSL) was calculated as the average velocity of motile sperm when measured in a straight line from the start point to the end point. Lateral amplitude (ALH) was calculated as the swing of the sperm head as it moved forward. Beat frequency (BCF) was calculated as the frequency of sperm head crossings relative to the sperm migration path. Straightness (STR) was calculated as how close to a straight line the average migration path of sperm was. Between 500 and 1000 sperm were analysed in each experiment.

### Embryo transfer

Embryos obtained by IVF (pronuclear fertilised oocytes and two-cell embryos) were transferred into the oviducts of pseudo-pregnant Crl:CD(SD) female rats^33^. Pseudo-pregnant rats were generated by mating female rats that were judged to be in proestrus by observing vaginal smears with vasectomised Crlj:CD(SD) male rats. Anaesthesia was performed using a mix of three types aesthetic agents administered at a dose of 0.5 mL per 100 g of body weight of the rats. Six to twelve fertilised oocytes were placed in one oviduct. After embryo transfer, an antagonist (antisedan, 150 µg/mL) was administered at a dose of 0.5 mL per 100 g of body weight of the rats. The number of live pups was counted after 22–23 days. The birth rate was calculated as the number of live pups divided by the number of embryos transferred and multiplied by 100.

### Cold transport of rat cauda epididymides

Cauda epididymides were collected from euthanised male rats (SD-Tg(CAG-EGFP)4Osb) at 14:00 h. The epididymides were transferred in a tube filled with Lifor containing 15% DMSO and 200 µg/mL quercetin and packed in a cold-transport kit using the same method as described above section of cold storage of cauda epididymides. The cold-transport kit was transported from Niigata University to Kumamoto University via cold transportation (temperature, 5.5–7.5 °C) by a shipping company. The transport kit arrived at Kumamoto University after 44 h and the transported epididymides were stored in a refrigerator at 4 °C prior to use. After 20 h storage, the sperm were collected at 06:00 h. At 09:30 h, oocytes were collected from euthanised female rats following superovulation treatment as described in the section above and used for IVF. Between 09:00 and 11:00 h, vaginal smears were confirmed and Crl:CD(SD) female rats in early proestrus were mated with vasectomised Crlj:CD(SD) male rats. The number of fertilised oocytes was counted at 15:00 h. The next day, plugged female rats were confirmed between 08:00 and 09:00 h and used as pseudo-pregnant rats for embryo transfer. Live pups were counted at 22–23 days after embryo transfer.

### Statistical analysis

Statistical analysis was performed using Prism version 8 (GaphPad Software) with arcsine transformation of percentage data, followed by analysis of variance and significant difference testing using Tukey or Dunnett test. Statistical significance was set at 5% or below the 1% level.
